# Steroid boost for sirtuin research

**DOI:** 10.18632/aging.100386

**Published:** 2011-09-18

**Authors:** Tobias Rumpf, Manfred Jung

**Affiliations:** ^1^ Institute of Pharmaceutical Sciences, Albert-Ludwigs-Universität Freiburg, Albertstr. 25, 79104 Freiburg, Germany

The posttranslational reversible acetylation on the ε-amino-group of lysines is an important switch in gene regulation and protein activity and has gained increasing interest in molecular biology and drug discovery in the last decade. One of the enzyme families that catalyze the cleavage of an acetyl group are the NAD+-dependent protein deacetylases, the sirtuins. The human sirtuins have seven members, Sirt1-7. Even though the biology of the sirtuins is far from being fully understood, there is already ample evidence that they may serve as future therapeutic targets. Their activity has especially been connected to processes in aging and hence, the field of potential applications for sirtuin modulators is very wide, ranging from neurodegenerative diseases to cancer to diabetes [1]. Some in vivo studies with sirtuin modulators, like the potential activator resveratrol or the inhibitor AGK2, show already great promise [2,3].

Several classes of sirtuin inhibitors with diverse chemical scaffolds have been published so far, but there is lack of highly specific inhibitors for only one of the seven sirtuin isoforms. Often only Sirt1 and 2 have been tested [4]. This lack of specific inhibitors may be partly because of the lack of high-resolution sirtuin-inhibitor x-ray structures but also due to the fact that the binding of the substrate is accompanied by a structural reshuffling of the substrate pocket [5]. Sirtuin isoforms are located in different cellular compartments and permeability of the compartment's membrane for the inhibitor may be a limiting factor, especially for the mitochondrial sirtuins Sirt3-5. For a selective inhibition of nuclear and cytoplasmatic sirtuins (Sirt1-2, 6-7) this might actually be favorable. Schlicker et al. now used virtual screening and docking methods [6], an approach that has already been successfully applied for the discovery of other sirtuin inhibitors [7], to identify new isoform-specific inhibitors. Two of the newly identified lead structures for the Sirt2-specific inhibitors contain a steroid scaffold and one of them is an ester of estradiol. The steroid scaffold brings along potential disadvantages due to possible interactions with steroid receptors.As there is ample knowledge in the literaure on modifications of the steroid scaffold, this could be used to eliminate such undesired side effects in further steroid-type sirtuin inhibitors. Besides the inhibition in vitro, the authors also showed the isoform-specific inhibition of Sirt2 in cell lysates as a more physiological environment but not in living cells.

This was due to the high cytotoxicity of the tested compounds but it was not clear if the cytotoxicity was a result of the Sirt2-inhibition. Nevertheless, the new findings should serve as a very good starting point for the identification of new potent and isoform-specific inhibitors. These are certainly needed for functional studies to shed light on the underlying cellular mechanisms for the different sirtuin isotypes and on their potential role as therapeutical targets or antitargets. The identification of the steroid scaffold as a sirtuin interacting structure is also very interesting for a more general point of view. It raises the question if physiological steroids may have an effect on the activity of sirtuins and further studies in that direction are awaited with great interest.
